# Weeds Enhance Insect Diversity and Abundance and May Improve Soil Conditions in Mango Cultivation of South Florida

**DOI:** 10.3390/insects14010065

**Published:** 2023-01-10

**Authors:** Blaire Kleiman, Suzanne Koptur

**Affiliations:** 1 Agroecology Program, Department of Earth and Environment, International Center for Tropical Botany, Institute of Environment, Florida International University, 11200 SW 8th St, Miami, FL 33199, USA; 2 Plant Ecology Lab, Department of Biology, International Center for Tropical Botany, Institute of Environment, Florida International University, 11200 SW 8th St, Miami, FL 33199, USA

**Keywords:** weeds, insects, mango, soil

## Abstract

**Simple Summary:**

When weeds are left in mango farms, especially native wildflowers, there is a potential they may provide pollen and nectar to increase beneficial insect abundance and diversity. We examined how weed presence affects insects on mango trees as well as soil health. We found that weeds significantly increased pollinating and parasitoid (natural pest control agents) insect abundance and diversity. There was no difference in predatory insects between treatments, and slightly more herbivorous insects on weedy mango trees. Pollinators in mango flowers and spiders were also greater on weedy mango trees. Soil conditions were significantly improved in soil carbon and pH by the presence of weeds. These results show that a tolerable level of weed species may benefit insect, plant, and soil biodiversity in farms which oftentimes have very little life on them.

**Abstract:**

This study examined if weeds could serve as insectary plants to increase beneficial insect abundance and diversity in mango cultivation in southern Florida. Additionally, we examined how weed presence affects mango tree soil health. We found that weeds significantly increased pollinating and parasitoid insect abundance and diversity. Eight insect orders and eighteen families were significantly more abundant on mango trees with weeds growing beneath them than those where weeds were removed. There was no difference in predatory insects between treatments, and slightly more herbivorous insects on weedy mango trees. Pollinating insects visiting mango flowers in the weed treatment were significantly greater, as well as spiders on weedy mango trees. However, there were more lacewings (Neuroptera) observed on the mango trees without weeds, and leaf chlorophyll in the old and new mango leaves was significantly greater, in the weed-free treatment. Soil conditions, however, significantly improved in soil carbon and a greater pH reduction in the presence of weeds, though weeds affected neither soil nitrogen, phosphorous, nor chlorophyll in productive green leaves. These results show that a tolerable level of selective weed species’ presence may benefit insect, plant, and soil biodiversity in farms. This is important in increasing production, sustainability, and biodiversity in agriculture, which otherwise may be deficient in non-crop life.

## 1. Introduction

There has been a surge of research on sustainable farming practices, and a push for conservation agriculture over the last half century [1]. Incorporating a diversity of plants as well as beneficial insects can be an important way to increase food production while reducing damaging chemical inputs such as fertilizers, herbicides, and pesticides on farms [2]. The presence of non-crop insectary plants, or plants to support beneficial insects, may provide help [3]. Weeds, or any wild non-crop plants growing where they are not wanted, may hold potential to serve as insectary plants in farms. They can provide floral and alternative prey resources for a wide array of beneficial insects: pollinators, parasitoids, and predators [4]. Utilizing insectary plants in farms is an integral part of sustainable farming [5] but using weeds as such is an emerging area of research [6,7].

Various hypotheses may be used to understand the interactions of weeds, crops, and insects, including concepts such as apparency, resource concentration, diversity/stability, and the “enemies” hypothesis [8]. These theories provide a context in which to examine the potential of weedy plants to benefit cropping systems. Studies have shown enhanced success of populations of beneficial parasitoid insects when weeds are present, as weeds provide floral resources that the beneficials use as adults to increase fecundity and extend the lifespan [9]. Pollinator populations may also benefit from weeds and have been shown to have a positive relationship with non-crop plants and crop pollination [10].

Vegetation surrounding farms and undisturbed habitats in agricultural landscapes is known to help sustain beneficial insects in farms [11]. However, the economic value of field margins to crop production has been little studied, and management of field borders to promote beneficial insects is not common. These insects may act as natural enemies, providing biological control of crop pests, as well as serve as pollinators, ensuring crop production. Weeds may provide alternative floral resources for pollinators, encouraging them to remain near the farm in the time between crop flowering events [12]. For crops dependent upon insect pollination, this could be extremely helpful.

Worldwide, nearly 35% of crops depend on pollinators [13]. Tropical crops, such as mango, can suffer pollination failure from habitat loss as natural areas supporting pollinating agents are eliminated or reduced [14]. Globally, the annual value of insect pollination exceeds US $195 billion to ~US $387 billion annually [15]. Natural pollinators increase the size of harvests, as well as their quality and stability, for 70% of important global crops [14].

There is a pollinator decline crisis in farms [16], as terrestrial insect abundance is estimated to decline by about 9% each decade [17]. Additionally, insect pollinators buffer and even benefit crop steadiness as the climate changes, becoming more important with increasing occurrences of heat waves [18]. Pollinators in an area are healthiest when there are 15 or more flowering plant species blooming collectively over an extended period [19]. Refuges with weeds may provide this continuous food supply to floral visitors. When supported by appropriate surroundings, native pollinators can provide free, complete, and even more efficient pollination services than the commonly used European honeybee (*Apis mellifera*) [20].

Parasitoid insects can serve as biological control of pests as they lay their eggs inside or on a host to ultimately kill. Weeds can provide nectar to adult female parasitoid wasps [10] and flies [21] and pest outbreaks may be less common due to increased mortality of pests due to parasitoids. The presence of weeds adapted to local environments has been shown to support parasitoids at equal rates to common insectary plants [6]. In situations where growing non-native insectary plants is difficult or costly, weeds may serve as insectary plants, supporting the nutrition and diet variety of parasitoids.

Weeds can also provide additional resources to parasitoids, other than nectar and pollen, such as oviposition or egg-laying sites. Many important economic pests may prefer to oviposit on weeds rather than the crop, whereas some parasitoids of pests may overwinter on weeds [22]. Similarly, vegetation surrounding mango trees may serve as refuge for parasitoid wasps of scales (Hemiptera: Coccoidea and Diaspididae), as well as fruit flies [23]. Serving as a reservoir of alternative prey and oviposition sites, weeds can support protection of crop yields by a healthy population of pest’s enemies.

Overall numbers of arthropods, including predators, can be enhanced by plant biomass provided by weeds. When studying weed–insect relationships of winter wheat, Smith et al. [24] found that herbivores had a greater positive relationship than predators with weed cover. However, total weed cover was positively related to insect predator abundance. The density and activity of predatory ground beetles has been found to be greater when there is more diversity and abundance of plants. Additionally, plant diversity within farms is found to greatly increase insect abundance as well as richness, when analyzing 60 different case studies [25]. In cultivated fields, weeds can contribute to biodiversity by hosting populations of granivorous carabid beetles via alternative weed seeds for them to consume [26]. In mango agroecosystems, weeds may serve as an appropriate habitat for predatory insects such as ants and beetles (Staphylinidae and Histeridae), important predators of crop-damaging *Anastrepha* fruit flies [23].

Mango (*Mangifera indica L.*) relies on successful insect pollination for quality fruit and their flowers are unspecialized to allow generalist pollination [27]. Managed pollinators such as honeybees are not effectual (e.g., Kevan 1999) [28] and are unsuccessful without additional pollinators [29]. Managed honeybees do not find mango flowers attractive [29], nor is hand pollination economically viable.

Mango flowers do not exhibit properties for wind pollination either morphologically or physiologically; for example, anthers have relatively low amounts of pollen grains (~200–300). Additionally, the stigma is less conspicuous when compared to flowers adapted to catch windborne pollen grains. Floral nectar for rewarding visitors also suggests entomophilous pollination of mango flowers [30].

Insect visitors to most frequently visit mango flowers are the insect orders Coleoptera, Diptera, Lepidoptera, and Hymenoptera, with dominant pollinators of mango being hymenopteran and dipteran [27]. Blow flies (Calliphoridae) are particularly effective pollinators of mango, with flower flies (Syrphidae) also reported as good pollinators of this order [31]. These flies are capable of transporting pollen over long distances, reproducing rapidly, and are attracted to mango flowers [32].

Native plants around the edges of sizable mango farms were found to increase both the abundance as well as the diversity of flower visitors to mango, while limiting the negative effects of isolation from pollinator habitats [33]. These increased visitor numbers produced greater mango fruit production, while native plant margins did not increase mango pests. Farmers commonly eradicate weedy vegetation to eliminate soil and pollinator competitors. However, a diverse array of flowers before, during, and after crops flower can improve, rather than harm, pollination of many crops, all flowering in unison [34]. Mango is a perennial, pollinator-dependent tree, making the creation of flower reserves potentially profitable with negligible competition, while improving yields in existing farmland [33].

Weedy vegetation has many interactions in agroecosystems, both for aboveground as well as belowground communities. While weeds and crops interact aboveground through insect visitors, they also interact belowground, mediated through soil microbial communities. In mango and other tropical fruit agriculture, tilling to remove weeds is often not feasible, and a no-till approach is practiced to not damage surface feeder roots [35]. Similarly, tropical fruit production has high turnover of plant organic matter from the warm wet climate.

Some plants may harbor soil bacteria and fungi that may either benefit or hurt other plants and the crop. For example, mycorrhizal fungi form symbiotic associations with some weeds and crops, benefiting plants by enhancing nutrient and moisture absorption and protection against disease [36]. Furthermore, some plants have an allelopathic root zone or rhizosphere, in which they directly inhibit the growth of other plants. Weed presence may be harmful, such as parasitic, allelopathic, and competition for crops. In some instances, agricultural weeds may hurt crops by competing for light, moisture, space, and nutrients. Potential competition may occur between weeds and mango trees for soil carbon, nitrogen, and phosphorous, with resulting diminished plant chlorophyll and negative effects on mango tree health.

**Figure 1:**
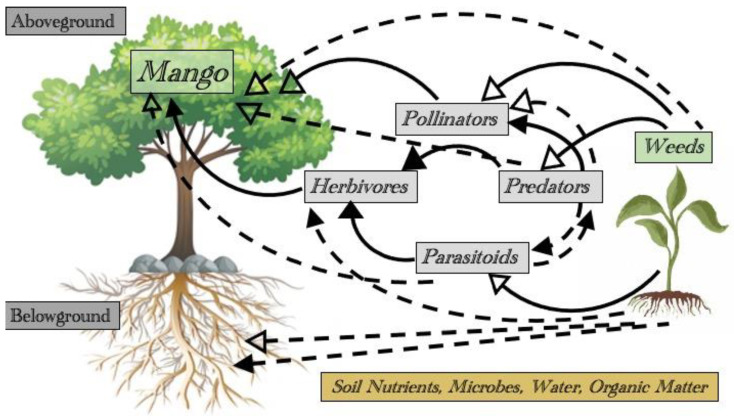
Potential interactions between weeds and mango trees, above and belowground. Direct interactions (solid line) and indirect interactions (dashed line); positive effects (open arrowhead), negative effects (solid arrowhead).

However, this is not always the case, as some weed species perform ecosystem services by protecting and restoring degraded soils [37]. Their roots provide habitat for beneficial microorganisms, add nutrients, food, and organic matter, and absorb and recycle nutrients which would otherwise leach out of the soil [38]. Additionally, they protect the soil surface from crusting, erosion, and evaporation. Further, decomposition of weeds can bring nutrients to the surface and add organic matter [39]. Weeds promote carbon cycling, which helps with water, nutrient, and organic matter retention [40]. Weed management, including appropriate control measures for invasive species causing economic loss, can be practiced, to allow the gains from beneficial weed species to be realized. These interactions provide promising methods for weed and insect management in sustainable farming (Figure 1).

The research objective is to examine increasing biodiversity using weeds as insectary plants to benefit the abundance as well as diversity of beneficial arthropods to increase sustainable mango crop production in South Florida. Our questions are:

(1) How does weed presence under mango trees affect the number and diversity of beneficial and pest arthropod on the mango trees?
(2) How do weeds impact soil conditions?

Our hypotheses:

(1) There will be a higher abundance and diversity of beneficial insect species on the mango trees with weeds than on the trees without weeds present.
(2) The presence of weeds may change properties of soil health beneath mango trees.

Support for these hypotheses will indicate that the presence of weeds is beneficial for mango cultivation. If the data do not support the hypotheses, this may indicate that weeds may have no effect, or a damaging effect, on mango insect populations, rather than providing resources to beneficial insects. Furthermore, if weeds reduce nutrient availability in the soil or otherwise worsen the substrate conditions for mango trees, their removal will also be indicated.

## 2. Materials and Methods

### 2.1. Site Description

The Redland farming area in Homestead has young soil with low organic matter, nutrient content, and is alkaline. The characteristics of the field site in Homestead, FL, USA, are gravelly loam calcareous soils with an average pH of 7.6, categorized as Krome Series by USDA NRCS [41], with high carbonate rock content. The mango trees at this experimental site are all mature 30–40-year-old trees with deep taproots. These trees contribute substantial leaf litter, adding organic matter and nitrogen to the soil as it decomposes, important for the organic poor calcareous soils of Homestead, FL. Homestead’s subtropical climate has two seasons, the “rainy season”, with hot, wet summers, and the “dry season” of a mild, dry winter. The average rainfall per year is 60 inches (~153 cm), with ample heat and sunshine all year long and little risk of frosts [42].

The experiment was located on a mango farm (~20 acres, 8 hectares, or ~0.1 km^2^) (25°29′42.9″ N 80°29′30″ W), using the variety “Keitt”. The farm is in a major agricultural area in South Florida. This large family farm practices ecologically oriented management and minimal chemical use in comparison to most mango farmers. No insecticides are used that might interfere with pollinators and natural biological control of pests. Additionally, US laws limit chemical applications to mango trees, such as outlawing Topsin (thiophanate-methyl), commonly used in other countries to treat the fungal infection mango malformation [43]. Fungicides were the only major chemicals applied by this farmer to control anthracnose, bacterial, and fungal pathogens. The fungicides applied were synthetic Bravo (chlorothalonil), biological Double Nickel 55 (*Bacillus amyloliquefaciens* strain D747), and organic OxiDate (hydrogen peroxide). Spraying was based on weather once flowering of mango had begun about every 1–4 weeks, depending on rain/humidity and continued until fruiting in July. When conditions were dry, fungicide spray was not needed, however once the rainy season began it was applied weekly. Micronutrients including zinc, iron, and manganese were applied before fruiting, and sulfur, potassium, and magnesium were applied in spring. Cow manure was applied instead of synthetic fertilizers, and mowing was performed by sickle bar around the mango trees in the weed-free treatment, so no herbicides were applied.

**Figure 2:**
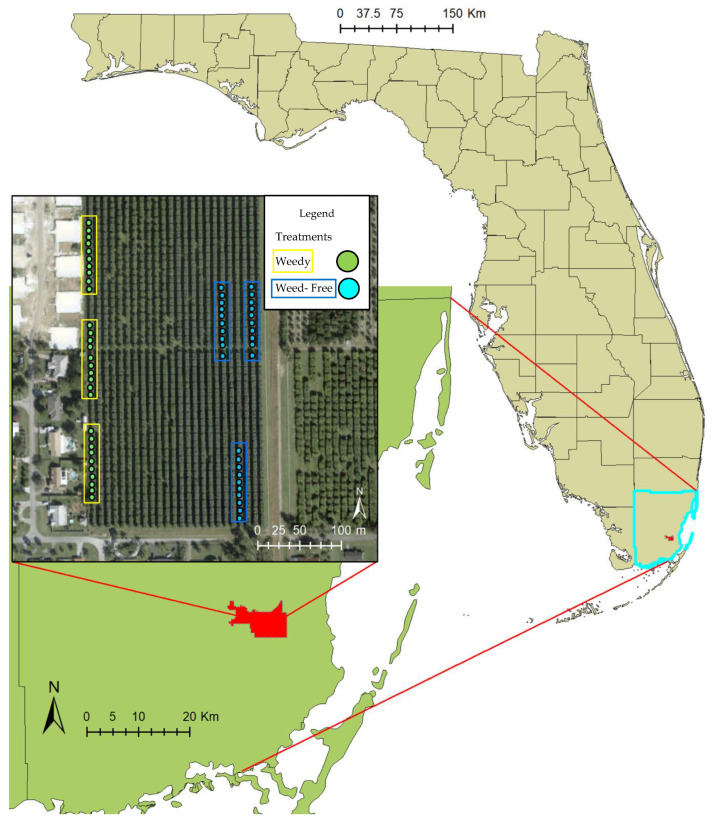
Location of the study site in southern Florida, USA, with location of farm and treatments within the mango grove indicating weedy and weed-free trees. Locations of plots were dictated by farm management practices.

This mango farm has 24 rows, each with 47 mango trees (1128 trees total), containing several varieties such as Keitt, Tommy, Florida Red, and Kent. All trees in this experiment were of the variety Keitt. Trees are evenly spaced in 20 × 20 feet (~6 × 6 m) breaks. Spacing between the rows is also ~20 feet (~6 m). The two treatments for the trees were weedy vs. weed-free. Each treatment had 30 trees with additional rows interspersed between (Figure 2).

The weed treatment allowed all weed growth around and between the mango trees, with each weed species identified (see list of weed species by Kleiman et al. [44]). Due to management requests by the farmer, trees for the weedy treatment were all designated on the same side of the farm, while the non-weedy trees were in three blocks (Figure 2). The area surrounding the weedy treatment is bordered by residential backyards, providing constraints from edge-effects and differing homeowners’ management practices on the weedy mango trees. The weed-free treatment is on the opposite edge of the farm, bordered by a railroad track and a neighboring farm. The weed-free treatment had the weeds removed beneath the trees with a string trimmer and mower, tools normally used by the farmer for removing vegetation beneath the mango trees.

### 2.2. Field Data Collection

Insects interacting with mango trees in this experiment were collected and recorded every week for ~six months, as in a recent study of effects of wildflowers on mango [33]. This period covered the eight-week flowering of the Keitt trees, as well as the entire mango flowering season, which runs from November to May. We conducted 5-min watch periods as we moved from tree to tree, recording insect interactions and collecting insects from all the 60 trees. These timed observations totaled 7500 min over 25 weeks.

The order each tree was visited was rotated each collection day for a wide sample across time per tree. We used close-focusing binoculars for observing insects in the canopy of trees as needed. Each insect on the tree and flowers were collected and recorded with representatives of taxon/type preserved for identification.

Insect specimens were collected if sight identification was not possible, and if they displayed notable behavior. Specimens were collected with an insect aspirator, hand net, or collection bag. We placed field-collected specimens in individual plastic containers which were stored in a freezer at 0 °C for later identification. Specimens were stored in ethanol vials or pinned and remain in the authors’ collection for eventual deposition in the Florida State Collection of Arthropods, in Gainesville, FL, USA.

### 2.3. Soil Analysis

We sampled soil at the beginning and end of data collection. A first sample was taken during the flowering stage of the mango trees on 23 February 2020, from 2 random trees per all sections of both treatments for 12 total samples. A second sample was taken at the end of data collection from the same trees after the harvest season concluded on 06/03/2020. Four samples were taken from a depth of 0–15 cm around each tree and combined to form a composite soil sample for each tree in the study. Subsamples of collected soil were oven-dried (30 °C), sieved in a 2 mm sieve, then ground for analyses at Florida International University’s Soil-Plant-Microbiology Laboratories (Miami, FL, USA). Measures of pH, chlorophyll, carbon, nitrogen, and phosphorous content were made to compare between treatments.

### 2.4. Soil pH

Readings of soil pH were taken to determine any changes in soil acidity caused by weeds, which might affect mango tree health, nutrient uptake, and growth. Soil slurries were made with a 3:1 deionized water to soil ratio, 4 g of distilled DI water to 2 g of wet soil, and pH readings were taken with an Orion 3 Star Benchtop pH meter (Thermo Fisher Scientific, Waltham, MA USA), following methods outlined by Miller and Kissel [45]. The ideal pH range for mango cultivation is between 4.5 and 7.5 pH, which is neutral to acidic soil, although mango trees do tolerate slightly alkaline soil [46].

### 2.5. Total Carbon and Nitrogen

Prepared soil samples had total carbon and total nitrogen analyzed by dry combustion with a Truspec carbon/nitrogen analyzer (LECO corporation, St. Joseph, MI, USA), using the AOAC official method 972.43 [47]. Both C and N elements were measured with gas combustion, which does not differentiate between organic and inorganic forms [48]. Total carbon and total nitrogen measured both the inorganic and organic forms of carbon and nitrogen in soil.

### 2.6. Total Phosphorus

Total phosphorous in soil samples was analyzed following the USEPA determination of soil phosphorus using the semi-automated colorimetry soil method 365.1 [49] in an AQ2 Discrete Analyzer (SEAL Analytical Inc., Southampton, UK). Total phosphorous was evaluated per sample following the methods and calculations of Sanford and Larson [50].

### 2.7. Chlorophyll Analysis

To compare and monitor mango tree health between treatments, the average leaf chlorophyll concentration of each plant was measured using a Soil-Plant Analyses Development (SPAD) 502 Plus Chlorophyll Meter (Konica Minolta, Inc., Tokyo, Japan), following methods by Uddling et al. [51]. A SPAD chlorophyll meter was used as a diagnostic tool to measure mango leaf chlorophyll as a proxy for tree nitrogen status during the flowering stage on 23 February 2020. Two random trees per section (twelve total) in both treatments were chosen for the non-invasive SPAD analysis. Three new growth leaves, three regular, and three old leaves were analyzed per tree, and obtained values were averaged.

### 2.8. Statistical Analyses

Descriptive analyses were conducted to describe variables of interest by treatment. These include standard deviations, means, proportions, and frequencies. A two-sample t-test was used to compare means between insect numbers. A chi-square test to see if the observed distribution of insects differs from the expected distribution was used. Multivariate tests such as Wilk’s Lamba, Pillai’s Trace, Roy’s Largest Root, and Hotelling’s Trace were analyzed for overall model significance, with following analyses using the simplified F-test comparison, adjusting for multiplicity.

Statistical analyses of soil results were performed for pH using a paired t-test and univariate analysis of variance (ANOVA). Soil carbon, nitrogen, and phosphorous were analyzed using an ANOVA and a paired t-test. Soil phosphorus was analyzed with a one-way ANOVA, t-test, and nonparametric tests. Leaf chlorophyll SPAD (Soil-Plant Analyses Development) results were analyzed using a univariate analysis of variance (ANOVA), with LSD, Bonferroni, and SNK post hoc tests. Age of mango trees was considered as a covariate; however, it was not significant. All associations or differences were considered significant at the alpha level of 0.05, following the adjustment for multiplicity when appropriate. All analyses were performed using SPSS software.

## 3. Results

### 3.1. Insects on Mango

Insects were identified and categorized broadly as “floral visitor, parasitoid, predator, or herbivore”, based on the dominant lifespan influences on the crop. A significant effect of treatment (weeds) was seen on mango floral visitors (F_1,57_ = 37.36, *p* < 0.0001), parasitoids (F_1,57_ = 40.95, *p* < 0.0001), and nearly between herbivores (F_1,57_ = 3.82, *p* = 0.056), with more of all categories on the weedy mango trees (Table 1). There was no significant difference in predatory insects (F_1,57_ = 2.16, *p* = 0.15) determined by a MANOVA.

#### 3.1.1. Insect Orders

Weedy mangos hosted substantially greater numbers of Hymenoptera than did weed-free trees (Table 2, F_1,57_ = 93.39, *p* < 0.0001). Additionally, there were significantly more Diptera (flies) (F_1,57_ = 8.35, *p* = 0.005), as well as Lepidoptera (Table 2), both potential mango pollinators, on the weedy mango trees (F_1,57_ = 3.97, *p* = 0.051).

The order Hemiptera had a significant difference (F_1,57_ = 11.22, *p* = 0.001), with more on weedy mango trees. Additionally, Coleoptera were significantly greater in the weed treatment (F_1,57_ = 16.53, *p* = 0.000).

Other insect orders collected—Orthoptera, Odonata, Ephemeroptera, and Collembola (Table 2)—did not differ significantly between treatments. Most members of these orders have pest or neutral tendencies. There was no significant difference in thrips, however there were slightly less in the weedy treatment (Table 2: Thysanoptera F_1,57_ = 1.33, *p* = 0.254).

Of the non-insects, mites (Acaroformes) were significantly greater in the weed treatment (F_1,57_ = 4.98, *p* = 0.03), though sample sizes were small. There were significantly more lizards and spiders as well in the weed treatment, with both acting as generalist predators of insects (Lacertilia, F_1,57_ = 13.51, *p* = 0.001; Aranae F_1,57_ = 6.06, *p* = 0.017).

#### 3.1.2. Lacewings

There were significantly more lacewing adults on the weed-free trees, compared to the weedy trees (F_1,57_ = 11.33, *p* < 0.001). Additionally, there were significantly more Chrysopidae (green lacewing) adults on the weed-free mango trees (Table 3, F_1,58_ = 15.3, *p* < 0.0001), as well as eggs (t_1,57_ = −6.9, *p* < 0.0001) and larvae, though not significantly so (t_1,20_ = −0.18, *p* = 0.86).

#### 3.1.3. Insect Families

Insect family observations were aggregated by individual mango trees across both treatments. These were then compared between the two treatments using a MANOVA. There was a total of 126 different insect families observed across both treatments, of which 21 insect families differed significantly when weeds were present (Table 3).

Many insect families known to have mango pollinators were more well-represented on mango trees with weeds present (Table 3), including Apidae (honeybees), Calliphoridae (blowflies), Muscidae (houseflies), and Syrphidae (hoverflies). Pollinating Lepidoptera were greater in the weedy treatment as well, including skippers (Hesperiidae) and gossamer-winged butterflies (Lycaenidae).

Other insects which visit flowers, such as Vespidae (wasps) and Chalcididae (parasitoid wasps), were also greater in numbers on the weedy mango trees (Table 3). Many additional beneficial families were more numerous on the weedy trees, including ants (Formicidae), ladybugs (Coccinellidae), and parasitoid wasps (Braconidae).

Some injurious families were also more numerous on weedy mango trees, however, such as scales (Coccidae), aphids (Aphididae), and moths (Geometridae). However, many other families which were more common on weedy mango trees likely had neutral effects on the trees, such as Chloropidae, Ephydridae, Sciaridae, and Straiomyidae (Table 3).

Alternatively, some insect families were significantly more abundant in the weed-free treatment, including Chironomidae (non-biting midges). While this insect family was seen in mango flowers in small numbers, it is not well-documented as an effective mango pollinator, or pest. Leafhoppers (Cicadellidae), however, were also greater in number in the weed-free treatment, though not significantly (Table 3).

Other animal predators (non-insects) appeared to be more abundant on weedy mango trees: Anolis lizards, voracious insect-eaters, were marginally more common on trees with weeds present underneath, though the difference was not significant (F_1,18_ = 1.13, *p* = 0.292). Two families of spider predators, however, were more often observed on weed-free mango trees, though the differences were also non-significant (Araneidae F_1,18_ = 1.79, *p* = 0.187; Salticidae F_1,18_ = 0.55, *p* = 0.46).

#### 3.1.4. Insect Interactions

The weedy mango trees had significantly more insects visiting and feeding in mango flowers (F_1,56_ = 45.93, *p* < 0.0001). Neither the presence of parasitized aphids, indicative of parasitoid activity (F_1,56_ = 0.53, *p* = 0.47), nor the activity of predatory insects feeding on prey was significantly different between treatments (F_1,56_ = 0.113, *p* = 0.74).

#### 3.1.5. Spider Interactions

There were significantly more spider webs (F_1,44_ = 27.74, *p* < 0.0001) and spiders in mango flowers hunting potential visitors on the mango trees with weeds than without (F_1,47_ = 22.27, *p* < 0.0001). There were significantly more spiders when weeds were present, with more spider (Aranae) adults in the weed treatment. We also observed more orb-weaver spiders (Araneidae) in the weed-free treatment, though this difference was not significant.

#### 3.1.6. Mango Diseases and Insect Damage

There were significantly more instances of sooty mold found on weedy mango trees compared to the weed-free trees, as determined by a MANOVA (F_1,56_ = 8.8, *p* = 0.004). There were more instances of anthracnose on the weed-free trees, however this was not significantly different and sample sizes were small (t_1,8_ = −0.63, *p* = 0.5). Similarly, instances of insects with mouths on or scraping mango leaves were recorded, as well as feeding on honeydew produced by hemipterans. Neither were statistically significant, however there were more instances of feeding on honeydew in the weed treatment, though not significant (t_1,26_ = 1.86, *p* = 0.074). Additionally, insects feeding on fruit (t = 0.714, *p* = 0.479) was not significant.

### 3.2. Soil

There was no significant difference in either carbon or nitrogen concentrations in the soil samples from beneath the weed and weed-free treatments for both sampling dates at the beginning and end of the study using an ANOVA. There was no statistically significant difference when comparing either carbon or nitrogen in the weed-free treatment at the beginning and end of the study (carbon: F_3,11_ = 1.311, *p* = 0.297, nitrogen: F_3,11_ = 0.59, *p* = 0.629). Soil nitrogen content varied between 0.4% and 0.6% but did not change during the duration of the experiment and was not different between weedy and weed-free plots (Appendix A, Table A1). The t-test comparison showed no difference when comparing nitrogen from the weed treatment at the beginning of the study and the end, however there was a significant difference in carbon (carbon: t_1,10_ = −2.84, *p* = 0.017, nitrogen: t_1,10_ = −1.33, *p* = 0.21). Carbon significantly increased in the weed treatment from a mean of 11.13 to 12.77 over the course of the study.

#### 3.2.1. Phosphorous

Total phosphorous in soil did not significantly differ between treatments, either at the beginning or end of the study, determined by a t-test and one-way ANOVA (beginning t_1,10_ = 0.68, *p* = 0.51; end t_1,11_ = 0.46, *p* = 0.65). Similarly, there was no difference in total phosphorous when comparing all soil samples (beginning + end) between treatments (Appendix A, Table A1), or when combining treatments and comparing all soil samples across time (F = 0.82, df = 1, 23, *p* = 0.375).

#### 3.2.2. Soil pH

Soil pH did not significantly differ between treatments (paired-samples t-test and univariate analysis of variance, F_1,25_ = 0.001, *p* = 0.975). However, when comparing treatment (weeds) and time (before and after samples), there was a significant difference. For both treatments, pH became more neutral over time (F_1,25_ = 23.71, *p* = <0.0001; univariate analysis of variance). While both treatments became more neutral over time, the weed treatment had a greater margin of reduction in soil pH.

#### 3.2.3. Chlorophyll Analysis

When comparing leaf chlorophyll across treatments and leaf age, there was a significant difference (Table 4, F_1,2_ = 3.7, *p* = 0.03) determined by a univariate ANOVA. Mango trees in the weed-free treatment had higher SPAD readings, indicating greater chlorophyll content, most dramatically in the old and new leaves. The mature green leaves in both treatments contained similar amounts of chlorophyll.

## 4. Discussion

This study indicates that weeds can serve as insectary plants to increase floral visitors and parasitoid abundance and diversity. Eight insect orders and eighteen families were significantly more abundant on mango trees with weeds growing beneath them than on mango trees that were weed-free. Added floral diversity provided by weeds increased numbers of some beneficial insects without negatively competing with the crop for soil nitrogen, phosphorous, and chlorophyll in productive leaves. Soil conditions were also significantly improved in soil carbon and pH.

We found more floral visitors on the weedy mango trees compared to weed-free, as well as insects’ feeding activity significantly greater in the weedy treatment mango flowers. Weeds may have provided added floral diversity that drew insect pollinators to visit less attractive mango flowers in proximity [30,52]. These results contribute to a growing number of crops who have been found to have weeds contribute potential pollinators, including oilseed rape [53], sunflowers [34], tomato [54], and alfalfa [55].

The weed treatment likely enhanced habitat diversity, providing nesting materials, floral, and prey resources, all necessary for the various life stages of Hymenoptera and other insects. Flower strips have also been shown to increase 56 butterfly species by 82% [56], of which weedy wildflowers may serve as host plants and nectar sources.

Parasitoids were more abundant in the weedy than the weed-free treatment. This could be due to adult insects needing floral nectar, and the flowering weeds growing below the fruit trees increases their survival, retention, pest suppression, and fecundity in farms [57,58]. The added diversity provided by weeds can provide not only floral resources such as nectar to adults, but alternative hosts and prey [22]. These resources may sustain parasitoids, and parasitoid abundance has been found to significantly decrease with distance from flowers [59]. Maintaining floral resources near crops may serve to boost their biological control of crop pests. However, potential interactions of parasitoids attacking pollinators may arise as these population dynamics often fluctuate in time and space [57].

Predators were somewhat greater in number in the weedy treatment, however not significantly. Previous studies have demonstrated that weedy field strips increase populations of predatory *Orius spp*. (Hemiptera: Anthocoridae), which were greater in areas with weedy field margins compared to weed-free areas [60]. Additionally, reduced tillage and increased proximity to semi-natural habitats has been found to increase predatory functional diversity [61] and abundance [62]. Weeds provide benefits of habitat heterogeneity to predatory insects, including pollen, nectar, egg-laying sites, shelter, and sources of prey, and enforce no-till agriculture [60]. This difference could be due to the enemies’ hypothesis, which states that predatory insects are more effective at controlling herbivore populations in diverse systems of vegetation than simple systems [63].

There were significantly more spiders and their webs as well as spiders observed in mango flowers hunting potential visitors on the mango with weeds rather than without. These results add weeds as a potential reservoir for spider conservation, alongside grasslands [64] and wildflower-sown islands in farms [65]. Spiders may prefer to reside and hunt in the weeds than the mango trees, especially when mango trees are not in bloom.

Interestingly, one order, Neuroptera, was more abundant on the weed-free mango trees, including the green lacewing, Chrysopidae. This beneficial insect is often released for biological control of crop pests, as their larval forms, known as “Aphid Lions”, are efficient natural predators of many pest species [66]. Their higher counts in the control trees could potentially be from the presence of their larval food source: soft-bodied invertebrates such as aphids, thrips, mites, mealy bugs, whiteflies, and even small caterpillars. Additionally, oviposition and site selection may have been easier for lacewings in the weed-free treatment in the absence of habitat complexity. Furthermore, there were fewer overall numbers of insects in the weed-free areas, allowing for less competition for egg-laying sites on the weed-free mango trees.

Overall, herbivore numbers were nearly significantly greater on weedy mango trees. This follows other studies in which increased farmland habitat heterogeneity increased herbivore species richness, evenness, and abundance [62]. This could pose an issue to mango trees should specialist mango-damaging herbivores be provisioned by weeds and proliferate in large numbers. Scales, or Coccidae, were the only herbivorous mango pest encountered in large numbers on the mango trees, however their presence rarely causes serious damage to mango in Florida. Additionally, we saw a high diversity of herbivores, with no one species of mango pest becoming critically damaging.

There were significantly more instances of sooty mold found on weedy mango trees compared to weed-free trees. This is possibly due to the increased presence of the plant-sucking hemipteran insects that excrete honeydew, which facilitates the development of sooty mold (Ascomycete fungi) [67]. Sooty mold affects the photosynthesis activity of leaves, negatively affecting plant health. Large numbers of adults and nymphs can feed on the sap of the tender parts of leaves, inflorescences, and fruit. This weakens inflorescences and affects fruit set, induces fruit drop, as well as facilitates sooty mold on valuable plant parts [68]. However, there were more instances of anthracnose on the weed-free trees, though this difference was not significant and sample sizes were small.

Certain weeds have been found to benefit crop soil by fixing nitrogen and bringing nutrients up into the topsoil [39]. However, we saw no difference when comparing nitrogen in the weed treatment at the beginning and end of the study. Carbon, though, significantly increased in the weed treatment over time. This could potentially indicate that the presence of weeds benefits carbon availability in the soil by added organic matter, retention, biological cycling, and decreased runoff of nutrients. Soil carbon is derived from soil organic matter such as decaying herbaceous weedy groundcover. Carbon improves soil quality by supporting soil structure, storing water and nutrients, and supporting vital soil microorganisms [69]. The high turnover and establishment of weedy vegetation may act as the progenitor for the soil carbon cycle.

Total phosphorous in soil between treatments did not differ significantly at the beginning or end of the study. Similarly, there was no difference in total phosphorous when comparing all soil samples (beginning + end) between treatments or when combining treatments and comparing all soil samples across time. This could mean that the presence of weeds does not impact soil phosphorous either positively or negatively, nor do weeds affect soil phosphorous over time. Soil phosphorous derives from weathering of minerals in the soil [70], in which there may have been no substantial difference between treatments over the course of the study.

Soil pH did not differ significantly between the treatments. For both treatments, though, pH became more neutral over time. The degree in reduction, however, was more dramatic in the weedy treatment. This could mean that as herbaceous vegetation grows and decomposes, adding organic matter to the soil, it becomes more acidic. The ideal pH range for mango cultivation is 4.5–7.5 pH, which is neutral to somewhat acidic soil; therefore, the greater reduction of pH to 7.7 for the weed treatment could mean weeds can help mango trees become closer to the ideal soil pH, and that the longer weeds are present may increase this effect. However, the effects of weeds on soil pH may not be the largest driving factor considering that both treatments saw a significant reduction over time.

Soils in South Florida are a mixture of marl, sand, and limestone rock, making soil alkaline with a pH of approximately 7.8–8.4 [71]. The high pH of this soil makes it difficult to retain nutrients in bioavailable forms for plants. Weeds may add biomass to the soil, influencing the ionic exchange capacity and causing a more favorable pH range. Intercropping systems in mango with groundcover plants have similarly been found to improve soil pH [72]. Understanding the possibility of weeds in improving South Florida soil pH can be especially valuable in mango production.

There was a significant interaction between treatment and leaf age on chlorophyll readings. Mango trees in the weed-free treatment had higher SPAD readings, indicating greater chlorophyll content, most dramatically in the old and new leaves. Additionally, there may be a potential relationship between the significantly greater levels of sooty mold in the weedy treatment and a reduction in leaf chlorophyll in the weedy treatment by reducing the photosynthesis ability of leaves. To compare, in soybeans, when infested with weeds for the entire growing season, there was a 21.42 reduction in the SPAD value and a 48.42% decrease in yield [73]. However, by increasing nitrogen levels, less of a reduction occurred by tolerance of soy to the presence of weeds. These results may indicate that, in this study on mango, there was an adequate amount of nitrogen to buffer for the presence of weeds, and that the only effects of weeds on leaf chlorophyll were seen in new and old leaves. Additionally, as mango is a perennial tree crop, it is less susceptible to competition with herbaceous weedy vegetation. Nitrogen management by farmers, then, can be used to buffer crop health while in the presence of weeds. Further, tree crops not limited by nitrogen may be best suited to decreased negative effects of chlorophyll competition by weeds.

Weeds here have proven to benefit soil carbon and pH for mango trees and had no effect on soil nitrogen, phosphorous, or chlorophyll in green leaves. However, a possible negative effect of weeds on leaf chlorophyll was seen in the decreased leaf content in the weed treatment, though only in new and old leaves. Considering the benefits of weeds in increasing resource diversity for beneficial insects, examination of the negative effects of weeds should be considered in a whole-farm context when comparing the pros and cons by farmers.

We found Calliphoridae (blowflies), Muscidae (houseflies), and Syrphidae (hoverflies) to be the most abundant non-bee flower visitors with the highest pollinator potential for fruit production (see Kleiman et al. for an in-depth discussion of pollinators and fruit yield results) [44]. Our study suggests non-honeybee insects to be more effective pollinators of mango than managed honeybees, contributing to other emergent studies with similar results looking at citrus [74], apple [75], cacao [76], and many other crops favoring non-bee insect pollinators, valued at US $1.2 billion worldwide [77].

This study illustrated the benefits of not eradicating weeds and showed the potential for increasing the production of mango, a widely cultivated and highly valued crop of Homestead, Florida, worth US $20.49 million a year in South Florida, and growing, with the current net profit from mango growing estimated at US $0.24 per pound or US $0.11 per kilo [78]. This study also supports the practice of some local mango farmers who leave selected attractive and beneficial weeds, such as *Bidens* sp., around their trees [79]. Additionally, there are many other potential insectary plants, including flowers such as sweet alyssum and buckwheat [5], or native herbaceous plants such as the Bahama Senna [80], which may provide alternatives for farmers to support beneficial insects.

Increasing societal worry regarding the health risks associated with herbicides is important [81], and this research may encourage more farmers to avoid the use of herbicides in food crop cultivation and pursue no-till agriculture. However, as we saw more hemipteran pests, sooty mold, and lower leaf chlorophyll in the weedy treatment, there is a possibility that weeds might lead some farmers to utilize more pesticides as a result. Weeds may not benefit every crop, especially if herbivores of the crop are supported by the weeds (they were not in our mango study). Additionally, weeds slow down the soil warming/drying process, which may help farmers buffer against the risk of growing tropical fruit in increasing temperatures [18,39]. Understanding the current ecological interactions on farms and ecosystem services provided by insects, such as pest control, soil formation, and pollination [82,83], can help guide future conservation efforts to benefit agricultural organism biodiversity and humans alike and protect against biodiversity loss on farms.

## 5. Conclusions

The added floral diversity of weeds increased beneficial insect abundance and diversity as well as improved some soil conditions for mango trees. However, possible confounding factors may have arisen considering the constraints of the design of our experiment, where weedy mangos were restricted to one side of the grove, and not spread across the farm. More extensive studies are needed to understand the network effects of various weeds and cropping systems across growing regions. Additionally, there may be beneficial aspects of removing weeds for farmers, such as reduced risks of biological contamination, farm aesthetics, and ease of access and mobility. The socio-environmental relationships of farmers and weeds, as well as an in-depth economic evaluation, need further examination in future research.

Increased pressure to conserve the global biodiversity of plants and insects is needed with the vulnerability of monoculture farms facing climate change. By increasing the diversity of plants and insects, farms become more resilient to extreme events, and therefore using native weeds adapted to the local environment supports a healthy and resilient farm landscape. Loss of functional biodiversity in agriculture is unsustainable, with serious threats for food production. Using weeds to increase habitat heterogeneity within crop cultivation may be one tool to enhance food production for growing populations.

## Figures and Tables

**Table 1:** Numbers of arthropods in different categories on mango, weedy vs. weed-free. Significant differences have *p*-values in bold, as shown by GLM multivariate analysis of variance (MANOVA). N = 30 trees for each treatment.

Type of Arthropod	Weed-Free ($\bar{x}$ ± SE)	Weedy ($\bar{x}$ ± SE)	F_1,57_, *p*
Flower Visitor	17.47 ± 1.63	31.59 ± 1.63	37.36, **<0.001**
Predator	59.27 ± 1.60	62.60 ± 1.60	2.16, 0.15
Herbivore	42.78 ± 0.95	45.42 ± 0.95	3.82, 0.056
Parasitoid	7.25 ± 0.74	14.01 ± 0.74	40.95, **<0.0001**

**Table 2:** Insect orders on mango, weedy vs. weed-free. Number of occurrences on each individual tree over the study duration, mean abundance and SE for each treatment, arranged by density ranking. Significant differences have *p*-values in bold, as shown by GLM MANOVA. N = 30 trees for each treatment.

Order	Weed-Free ($\bar{x}$ ± SE)	Weedy ($\bar{x}$ ± SE)	F_1,57_, *p*
Diptera	58.4 ± 2.5	68.5 ± 2.5	8.35, **0.005**
Hemiptera	44.0 ± 1.0	48.7 ± 1.0	11.22, **0.001**
Hymenoptera	9.0 ± 0.9	21.2 ± 0.9	93.39, **0.000**
Lepidoptera	7.5 ± 0.7	9.6 ± 0.7	3.97, **0.051**
Thysanoptera	7.8 ± 0.5	7.1 ± 0.5	1.33, 0.254
Neuroptera	6.5 ± 0.5	4.3 ± 0.5	11.33, **0.001**
Collembola	4.1 ± 0.3	4.1 ± 0.3	0.01, 0.925
Odonata	2.0 ± 0.3	2.2 ± 0.3	0.35, 0.55
Coleoptera	0.7 ± 0.2	2.1 ± 0.2	16.53, **0.000**
Orthoptera	0.2 ± 0.1	0.3 ± 0.1	0.12, 0.73

**Table 3:** Average number of individuals of insect families on individual mango trees with/without weeds, arranged by order and abundance. Significant differences have *p*-values in bold, as shown by GLM MANOVA. N = 30 trees for each treatment.

Order/Family	Weed-Free ($\bar{x}$ ± SE)	Weedy ($\bar{x}$ ± SE)	F_1,57_, *p*
**Hemiptera**/Coccidae	37.3 ± 0.8	39.8 ± 0.8	4.749, **0.034**
Aphididae	2.1 ± 0.4	3.3 ± 0.4	4.569, **0.037**
Cicadellidae	2.3 ± 0.3	1.7 ± 0.3	2.469, 0.122
Aleyrodidae	0.9 ± 0.2	1.1 ± 0.2	0.613, 0.437
Flatidae	1.0 ± 0.2	1.0 ± 0.2	0.003, 0.958
Pseudococcidae	0.4 ± 0.1	0.5 ± 0.1	0.214, 0.645
**Diptera**/Dolichopodidae	19.7 ± 0.5	19.7 ± 0.5	0.001, 0.975
Muscidae	4.8 ± 0.7	8.7 ± 0.7	17.074, **0.000**
Syrphidae	4.5 ± 0.5	7.0 ± 0.4	15.277, **0.000**
Chironomidae	6.0 ± 0.4	4.6 ± 0.4	5.056, **0.028**
Tephritidae	3.6 ± 0.4	4.2 ± 0.4	1.089, 0.301
Calliphoridae	2.6 ± 0.4	4.0 ± 0.4	6.157, **0.016**
Sarcophagidae	3.7 ± 0.4	3.6 ± 0.4	0.074, 0.786
Ephydridae	1.8 ± 0.3	2.8 ± 0.3	6.994, **0.011**
Chloropidae	1.6 ± 0.3	2.7 ± 0.3	7.345, **0.009**
Phoridae	1.5 ± 0.2	1.9 ± 0.2	1.600, 0.211
Drosophilidae	1.3 ± 0.2	0.9 ± 0.2	1.96, 0.167
Mycetophillidae	0.8 ± 0.2	0.4 ± 0.2	2.537, 0.117
Sciaridae	0.3 ± 0.1	0.7 ± 0.1	5.150, **0.027**
Anisopodidae	0.6 ± 0.1	0.6 ± 0.1	0.007, 0.932
Stratiomyidae	0.1 ± 0.1	0.3 ± 0.1	5.097, **0.028**
**Neuroptera**/Chrysopidae	6.6 ± 0.4	4.3 ± 0.4	15.304, **0.000**
**Hymenoptera**/Apidae	1.9 ± 0.4	4.2 ± 0.4	19.075, **0.000**
Formicidae	0.3 ± 0.4	2.23 ± 0.4	11.723, **0.001**
Chalcididae	0.4 ± 0.2	1.4 ± 0.2	11.522, **0.001**
Vespidae	0.2 ± 0.2	1.1 ± 0.2	9.762, **0.003**
Ichneumonidae	0.7 ± 0.1	1.0 ± 0.1	1.727, 0.194
Braconidae	0.1 ± 0.1	0.8 ± 0.1	31.024, **0.000**
**Odonata**/Anisoptera	1.9 ± 0.3	1.9 ± 0.3	0.003, 0.958
**Lepidoptera**/Lycaenidae	1.4 ± 0.2	2.1 ± 0.2	4.854, **0.032**
Hesperiidae	0.5 ± 0.2	1.1 ± 0.2	8.443, **0.005**
Geometridae	0.0 ± 0.1	0.6 ± 0.1	10.033, **0.002**
Nymphalidae	0.3 ± 0.2	0.4 ± 0.2	0.066, 0.799
Pieridae	0.3 ± 0.1	0.3 ± 0.1	0.009, 0.924
**Coleoptera**/Coccinellidae	0.1 ± 0.1	1.1 ± 0.1	24.782, **0.000**
Scarabidae	0.1 ± 0.1	0.3 ± 0.1	2.880, 0.095

**Table 4:** SPAD chlorophyll readings for mango leaves, weeds vs. weed-free, total chlorophyll per unit leaf area (nmol/cm^2^). Univariate ANOVA comparisons. Letter superscripts indicate differences in mean SPAD readings within and between treatments.

Leaf Age	Leaf Chlorophyll Concentration on Trees in	
	Weedy Plots (N = 18 All)	Weed-Free Plots (N)
New	16.9 + 2.4 ^a^	36.6 + 5.1 ^b^ 22
Mature	54.4 + 1.9 ^c^	56.4 + 3.1 ^c^ 16
Old	23.0 + 3.1 ^a^	42.8 + 4.6 ^b^ 16

## Data Availability

Data to be deposited in the FIU Research Data Portal, a publicly accessible repository: https://doi.org/10.34703/gzx1-9v95/LB64KU (accessed on 5 January 2023).

## Appendix A

**Table A1:** Mean levels (±SE) of soil nitrogen (%), carbon (%), and phosphorous (mg g^−1^ dry weight), weedy or weed-free trees, ANOVA. N = 12 trees: 6 weedy, 6 weed-Free.

Nutrient	Weed-Free ($\bar{x}$ ± SE)	Weedy ($\bar{x}$ ± SE)	F_3,11_, *p*
**Nitrogen**			0.59, 0.629
Beginning	0.5 ± 0.1	0.4 ± 0.1	
End	0.6 ± 0.1	0.6 ± 0.1	
**Carbon**			1.311, 0.297
Beginning	12.3 ± 0.8	11.1 ± 0.2	
End	12.7 ± 0.8	12.8 ± 0.5	
**Phosphorous**			0.465, 0.511
Beginning	3.4 + 0.9	2.7 + 0.6	
End	4.0 + 0.7	3.5 + 0.9	
